# Who does Red Bull give wings to? Sensation seeking moderates sensitivity to subliminal advertisement

**DOI:** 10.3389/fpsyg.2015.00825

**Published:** 2015-06-19

**Authors:** Gaëlle M. Bustin, Daniel N. Jones, Michel Hansenne, Jordi Quoidbach

**Affiliations:** ^1^Department of Economics and Business, Universitat Pompeu FabraBarcelona, Spain; ^2^Department of Psychology, University of TexasEl Paso, TX, USA; ^3^Department of Psychology: Cognition and Behavior, University of LiègeLiège, Belgium

**Keywords:** subliminal priming, advertisement, personality, sensation seeking, consumer psychology

## Abstract

This study assessed whether subliminal priming of a brand name of a drink can affect people’s choices for the primed brand, and whether this effect is moderated by personality traits. Participants with different levels of sensation seeking were presented subliminally with the words Red Bull or Lde Ublr. Results revealed that being exposed to Red Bull lead on average to small increases in participants’ preferences for the primed brand. However, this effect was twice as strong for participants high in sensation seeking and did not occur for participants low in sensation seeking. Going beyond previous research showing that situational factors (e.g., thirst, fatigue…) can increase people’s sensitivity to subliminal advertisement, our results suggest that some dispositional factors could have the same potentiating effect. These findings highlight the necessity of taking personality into account in non-conscious persuasion research.

## Introduction

Can people’s choices be affected subliminally? Since the 1950s, there have been persistent myths about the efficacy of persuading others without their knowledge. In 1957, James Vicary, a market researcher from New Jersey, claimed that flashing messages encouraging popcorn eating and drinking Coca-Cola raised vendor sales. Although Vicary later admitted to fabricating the data, belief in the power of subliminal persuasion persists. For example, in 2000, a Republican support group ran an advertisement, which briefly highlighted the word “rats” in “Democrats,” in what appeared to be a subliminal persuasion attempt. The backlash was tremendous, leading the ad to be taken down. Similarly, McDonalds was accused of flashing the company slogan “I’m lovin’ it,” together with a hamburger in a single frame during a popular TV cooking show. About 75% of Americans have heard of subliminal messages (Rogers and Smith, 1993) and a staggering 62% of all U.S. adults believe that they are continuously being assaulted with subliminal messages (Haberstroh, 1994).

But can one, in light of the current scientific evidence, draw any conclusions regarding the effectiveness of subliminal persuasion techniques? Many early studies suggest that subliminal messages can indeed affect people’s choices (Caccavale et al., 1982; Kilbourne et al., 1985; Gable et al., 1987). However, meta-analytic approaches indicate that the effect sizes of such subliminal persuasion attempts are weak (around *r* = 0.06; see Trappey, 1996). More recently, however, experimental studies have shown that subliminal priming can meaningfully influence consumer behavior, provided that brand or product being primed is relevant to the consumer’s goals and needs. For example, Karremans et al. (2006) demonstrated that the brand name “Lipton Ice” presented subliminally could increase participants’ intention to drink this specific brand, but only when participants were thirsty (e.g., when given salty food before exposure to the prime). Likewise, Bermeitinger et al. (2009) found that participants performing a computer game that subliminally primed the pill brand, “dextrose” consumed more pills of the primed brand, but only when participants were tired. More recently, Veltkamp et al. (2011) used a subliminal evaluative conditioning method in which they paired consumer behavior with positive traits (e.g., drinking + good) below conscious awareness. The authors found that non-thirsty individuals, for whom the concept of drinking had been subliminally paired with positive words, consumed more water than did individuals in the neutral conditioning condition. Thus, in addition to a direct effect of deprivation (e.g., thirst, fatigue), subliminal conditioning can motivate individuals as if they were actually deprived.

Taken together, the aforementioned studies show that situational factors can increase people’s sensitivity to subliminal advertisement such that subliminal messages are more effective on those who are currently craving a particular product when compared to those who are not. Here, we propose that dispositional factors could have the same effect. Research has shown that individuals vary in their dispositions toward experiencing specific goals and needs. For example, Kaiser and Ozer (1994) found that Agreeableness was related to affiliative strivings and that Conscientiousness was associated with achievement-related strivings. Likewise, Roberts and Robins (2000) examined the relationship between major life goals and personality traits, finding that Extraversion was positively associated with political aspirations and Agreeableness was positively associated with social goals. Moreover, personality traits have been linked to consumer choice. For example, sensation seeking is associated with a preference for caffeinated drinks (Jones and Lejuez, 2005), whereas extraversion and openness are positively related to hedonic product value (Matzler et al., 2006). In sum, personality traits are typically associated with enduring propensity toward different goals and subliminal messages that align with people’s dispositional tendencies should thus be more effective.

We believe that examining personality moderation in subliminal processing is important for two reasons. First, the effect size of subliminal priming has been shown to be relatively small (see Trappey, 1996). Part of the reason could be that, depending on their traits, some people are sensitive to some subliminal messages but not to others, lessening the average effect size of subliminal priming. Second, exploring the relationship between personality and the perception of subliminal stimuli might provide valuable insight to design more effective tailored subliminal messages. As an initial test of whether personality moderates consumers’ sensitivity to subliminal advertisement, we conducted a study examining the link between Sensation Seeking and sensitivity to “Red Bull” primes. Traits such as extraversion, exploration, and novelty seeking are considered fundamental aspects of personality (Zuckerman et al., 1993), which are central to sensation seeking (Zuckerman, 1990). Sensation seeking reflects a tendency toward exploratory excitability in response to novelty, impulsivity, and is associated with sensitivity in the dopaminergic system (Zuckerman, 1979, 1994; Bódi et al., 2009). High sensation seeking individuals are characterized as risk-takers, whereas low sensation seeking individuals are arousal avoiders (Zuckerman, 1990). We chose to prime the brand *Red Bull* because it resonates with these concepts of excitation and risk-taking. In fact, a positive association between energy drink consumption and sensation seeking has been shown in several studies (Jones and Lejuez, 2005; Arria et al., 2010). Thus, as situational factor like thirst can increase people’s sensitivity to a goal-relevant subliminally primed refreshing drink (i.e., Lipton Ice Tea), we tested whether a dispositional factor like sensation seeking can increase people’s sensitivity to a goal-relevant subliminally primed energetic drink (i.e., Red Bull). Specifically, we hypothesized that priming Red Bull would affect participants’ intention to consume the energy drink. We additionally hypothesized that this effect would be particularly pronounced for those high in sensation seeking.

## Materials and Methods

### Participants

Our initial sample was composed of 160 American adults recruited through Amazon’s Mechanical Turk (MTurk; see Paolacci et al., 2010; Buhrmester et al., 2011 for recent reviews of the strengths of MTurk relative to other online and oﬄine data collection methods). From this initial sample, 13 participants failed to complete the study until the end and seven participants were removed from the analyses because they failed to pass validity check items (which are detailed in the Section “Procedure” below). These attrition and non-compliance rates are common for MTurk samples, and such cleaning is required for accurate samples from MTurk (Crump et al., 2013). Thus, the final sample was composed of 140 participants (66% female, *M*_age_ = 36.5 years, SD = 12.8). The experiment was approved by the local ethics committee and all participants provided informed consent.

### Procedure

Participants first completed a measure of sensation seeking and were then randomly assigned to an experimental or a control condition. Following Karremans et al. (2006), participants were told that they were going to participate in a visual detection task examining their ability to detect small deviances. In fact, this task was designed to subliminally prime half of our participants with the word “Red Bull,” and the other half with a control word, containing the same letters (i.e., Lde Ublr). Instructions stated that in this task a string of upper case “Q” (QQQQQQQQQ) would be briefly presented 30 times in the center of the screen. Every now and then, a lower case “q” would appear in a string of capital “Q” (e.g., QQQQQQqQQ). At the end of the 30 trials, participants were asked to indicate how many small q were presented.

Each trial consisted of the following sequence: first, a fixation cross appeared on the screen for 500 ms. Immediately after the cross disappeared, the words “Red Bull” (in the experimental condition) or the non-words “Lde Ublr” (in the control condition) was presented for 33 ms, immediately followed by the string of Q’s. The string of Q’s was presented for 300 ms and served to mask the prime.

After completion of the visual detection task, participants were asked to indicate their intention to drink “Red Bull” and their level of thirst. Lastly, as a validity check for the intended subliminal nature of the prime, we first asked participants whether they had seen anything unusual during the visual detection task on an open-ended response format. We then explicitly asked participants whether they had seen the word “Red Bull” flashing on the screen.

### Measures

#### Sensation Seeking

We measured Sensation Seeking with the well-validated Brief Sensation Seeking Scale (BSSS, Hoyle et al., 2002), which includes eight items rated on a scale from 1 (*strongly disagree*) to 4 (*strongly agree*). Examples of items include “*I would like to take off on a trip with no pre-planned routes or timetables*” and “*I would love to have new and exciting experiences, even if they are illegal*” (α = 0.87).

#### Intention to Drink Red Bull

Participants intention to drink Red Bull was measured with one item: “*If you had to stay up late, how likely would you order Red Bull*,” on a scale ranging from 1 (*very unlikely*) to 7 (*very likely*). We created this item to be as conceptually similar as possible to Karremans et al. (2006)’s item (i.e., “*If you would sit on a terrace now, how likely that you would order Lipton Ice”*), while being relevant to an energy drink. Participants also answered the same item with regard to intention to drink Coca-Cola and coffee.

#### Thirst

We measured participants’ levels of thirst with two items (“*How thirsty are you at this moment?*” and “*How much do you feel the need to drink at this moment?*”; α = 0.95).

## Results

### Manipulation Check

Because our study was conducted online, it was highly likely that participants used different computers, operating systems, and Internet platforms. Thus, we could not assess nor precisely control the exact timing of the subliminal stimulus. In order to ensure that participants did not accurately identify the stimuli (and therefore was not consciously perceived), we first examined responses to the validity check items.

Results showed that three participants reported having seen the words “Red Bull” on the open-ended question and four participants reported having seen the words Red Bull when we explicitly asked them. Those participants were removed from the analyses.

### Main Effect of Subliminal Stimulation

To test whether subliminal exposure to “Red Bull” positively affected participants’ intention to drink this brand of energy drink, we first compared participants’ intention to drink Red Bull in the experimental group with participants’ intention to drink Red Bull in the control group. In line with previous research, we found a small significant difference on average between the two groups, with participants primed with the brand “Red Bull” reporting higher desire to drink Red Bull (*M* = 3.41, SD = 2.36, 95% CI [2.84, 4.00]) than participants primed with the non-word “Lde Ublr” (*M* = 2.57, SD = 2.00, 95% CI [2.11, 3.00]), *t*(138) = 2.28, *p* = 0.02, *r* = 0.19.

### Moderating Effect of Sensation Seeking

**FIGURE 1:**
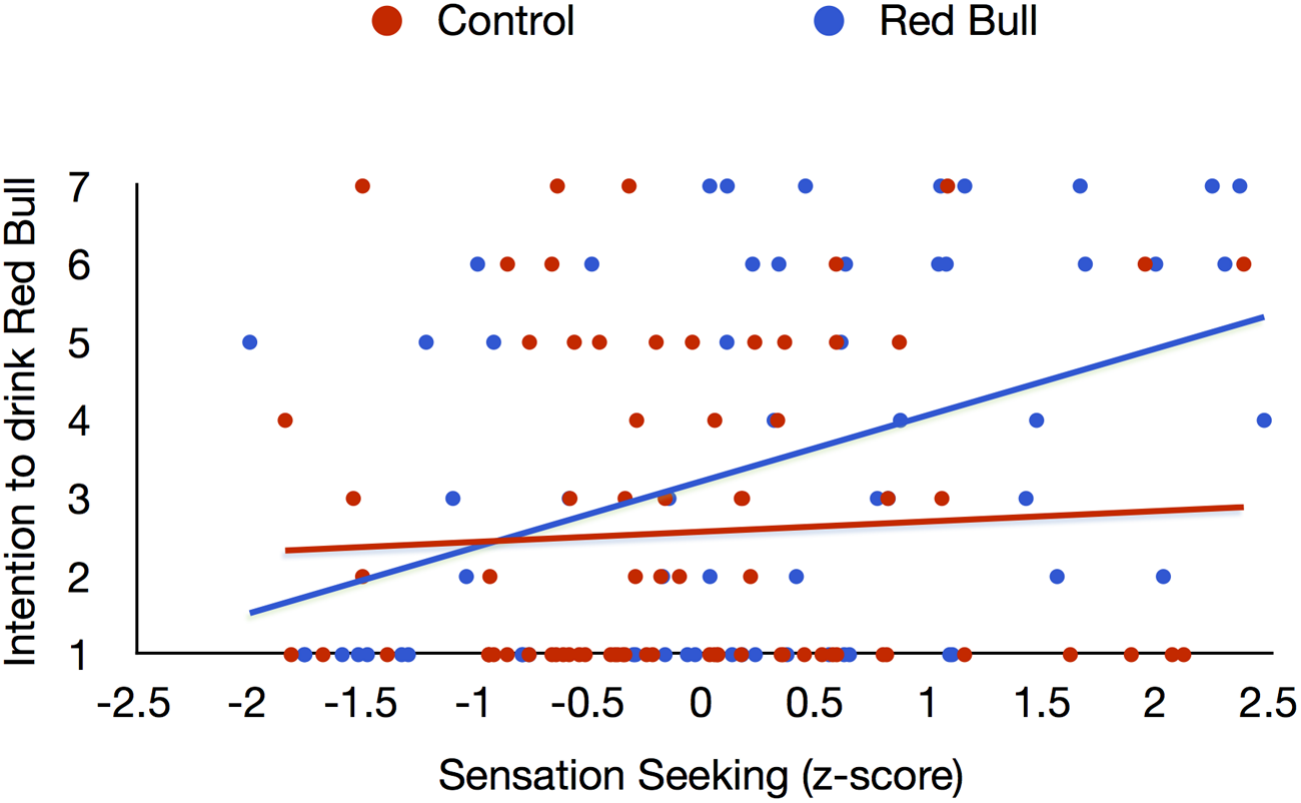
**Effects of subliminal exposure to the words Red Bull on intention to drink Red Bull depending on participants’ level of sensation seeking**.

To test whether participants’ levels of sensation seeking would moderate the effectiveness of the subliminal advertisement, we entered condition (coded 1 for the experimental “Red Bull” condition and –1 for the control “Lde Ublr” condition), participants’ standardized sensation seeking scores, and their interaction into a regression predicting intention to drink Red Bull. Results revealed a significant effect of sensation seeking (β = 0.23, *t* = 2.79, *p* = 0.006, *r_p_* = 0.23), a marginally significant effect of condition (β = 0.16, *t* = 1.96, *p* = 0.05, *r_p_* = 0.17), and a significant Condition × Sensation Seeking interaction (β = 0.17, *t* = 2.04, *p* = 0.04, *r_p_* = 0.17), depicted in **Figure 1**. To probe this interaction, we compared participants’ intention to drink in the experimental vs. control conditions at different levels of sensation seeking. Participants high in sensation seeking (i.e., 1 SD above the mean) presented with Red Bull were more likely to desire this brand than participants high in sensation seeking exposed to the control prime, *t* = 2.82, *p* < 0.01, *r_p_* = 0.24. In contrast, the tendency to order Red Bull did not differ between experimental conditions for participants low in sensation seeking (i.e., 1 SD below the mean), *t* = -0.04, *p* = 0.97, *r_p_* = -0.00.

As can be seen in **Figure 1**, there were more participants with very high scores of sensation seeking in the experimental group than in the control group. Although the mean level difference in sensation seeking between the two groups did not reach statistical significance, *t*(138) = 1.59, *p* = 0.11, we tested whether our results would hold by matching participants in the two groups on their level of sensation seeking. Specifically, we paired participants from the experimental group with participants in the control group that had the exact same level of sensation seeking. Participants in the experimental group that could not be matched with a control participant were discarded. When there more control participants than experimental participants at a given level of sensation seeking (i.e., when one participant in the experimental group could potentially be matched with several controls, we took the average score of intention to drink Red Bull of these control participants). Providing further support for our findings, a regression predicting intention to drink Red Bull from condition, sensation seeking, and their interaction in this new sample of perfectly matched individuals (*N* = 80) revealed a significant main effect of condition (β = 0.21, *t* = 2.17, *p* = 0.03, *r_p_* = 0.24), no significant effect of sensation seeking (β = 0.16, *t* = 1.17, *p* = 0.25, *r_p_* = 0.13) and a significant interaction (β = 0.37, *t* = 2.70, *p* < 0.01, *r_p_* = 0.30).

Were high sensation-seeking participants more likely to drink caffeinated drinks in general? A regression predicting intention to drink Coca-Cola from condition, sensation seeking, and their interaction revealed no significant main effect of condition (β = 0.03, *t* = 0.38, *p* = 0.71, *r_p_* = 0.03), no significant effect of sensation seeking (β = -0.06, *t* = -0.66, *p* = 0.51, *r_p_* = -0.06) and no significant interaction (β = 0.05, *t* = 0.58, *p* = 0.56, *r_p_* = 0.05). A similar regression predicting intention to drink coffee revealed a marginal trend for the main effect of condition (β = -0.16, *t* = -1.91, *p* = 0.06, *r_p_* = -0.16) with participants in the experimental condition reporting less desire for coffee, no significant effect of sensation seeking (β = 0.02, *t* = 0.21, *p* = 0.83, *r_p_* = 0.02) and no significant interaction (β = -0.02, *t* = -0.22, *p* = 0.83, *r_p_* = -0.02).

Were high sensation-seeking participants simply thirstier than other participants, which would explain as in Karremans et al. (2006)’s study that the prime was more effective for them? There was no correlation between thirst and sensation seeking (*r* = 0.00, *p* = 0.97). Moreover, including thirst as a covariate did not change the overall pattern of the results. Specifically, the Sensation Seeking × Condition interaction was still significant, β = 0.17, *t* = 2.04, *p* = 0.04, *r_p_* = 0.17 for intention to drink Red Bull, but not significant for intention to drink Coca-Cola, β = 0.05, *t* = 0.61, *p* = 0.55, *r_p_* = 0.05 and coffee, β = -0.02, *t* = -0.21, *p* = 0.83, *r_p_* = -0.02.

## Discussion

The dominant research question in subliminal persuasion so far has been to examine *whether* and *when* subliminal advertising works. The present study addresses for the first time another equally crucial question: “For *whom* does subliminal advertising work?” Our research provides initial evidence that the effectiveness of subliminal persuasion attempts can be moderated by personal dispositions. We found that exposing individuals subliminally to the brand name of Red Bull meaningfully increased intention to drink Red Bull for participants that were high in sensation seeking, whereas subliminal priming had no effect on participants that were low in sensation seeking.

Going beyond previous research showing that people’s sensitivity to subliminal advertisement depends on situational factors (Karremans et al., 2006; Bermeitinger et al., 2009; Veltkamp et al., 2011), the present study demonstrates that people’s sensitivity to subliminal advertisement can also depend on dispositional factors. These findings dovetail with research showing that the way people respond to various non-conscious stimulations can be moderated by individual differences. For example, Yoshino et al. (2005) showed that individuals high in novelty seeking showed higher skin conductance responses for subliminal positive and negative emotional stimuli compared to individuals low in novelty seeking. Likewise, Bustin et al. (2012) exposed participants to a cognitive updating task where they could gain either 5 cents or 1 Euro on every trial, and found that participants low in novelty seeking performed better on subliminal trials of high- vs. low-reward. Finally, research in the psychoanalytic tradition have demonstrated that individual differences such as anxiety, social desirability, and personal preferences can interact with priming effect with very stringent subliminal criteria, where all conscious influence is excluded (Snodgrass et al., 1993; Klein Villa et al., 2006; Bazan and Snodgrass, 2012).

Taken together, these findings highlight the importance of taking personality into account if one wants to estimate the real effect size of subliminal stimulation. As previously mentioned, meta-analytical work by Trappey (1996) found that the average effect size of subliminal stimulation is around *r* = 0.06, which according to Cohen’s guidelines, can be qualified as “weak” (Cohen, 1988, 1992). Our research might provide a potential explanation for the apparent weakness of these effects: in most previous work, personality has been neglected. In the present work, the percentage of explained variance doubles when looking at high novelty seekers rather than the whole sample (from 3 to 6%). Our results suggest that subliminal stimulation can be of practical significance, at least when targeted at specific personality-based subgroups of individuals.

Although the present research contributes in several ways, our findings should be considered preliminary and more research is certainly needed before one can draw general conclusions. In particular, future studies should document the precise pathways by which personal dispositions moderate the effect of subliminal advertisement, and subliminal primes in general. Previous work has shown that subliminal priming is especially effective when the prime is goal-relevant, that is, when the prime is related to something people need or want to achieve (Strahan et al., 2002; Winkielman et al., 2005; Karremans et al., 2006; Veltkamp et al., 2008). Based on this idea, we proposed that a similar logic is at play when it comes to personality: people differ in their sensitivity to subliminal stimuli because, depending on their traits, some primes are more relevant to them and more likely to capture their (unconscious) attention. According to this perspective, high sensation seeking people would be influenced by an energy-drink or a sports car prime but probably not by a retirement saving plan prime, whereas the opposite might be true for individuals high in conscientiousness. We note, however, that it is also possible that high sensation seeking participants could more effectively imagine the situation “If you had to stay up late,” and as a consequence, Red Bull was more relevant to them. According to this alternative perspective, priming other types of caffeinated drinks (e.g., coffee) to people high in sensation seeking—even if they don’t intrinsically value what the brand stand for—should lead to the same results. Finally, it is possible that sensation seeking is associated with perceptual differences in the way people process subliminal information in general. According to this perspective, people high in sensation seeking would have a strong reaction to any subliminal advertisement from Red Bull to brands of laundry detergent. Future research is needed to disentangle these three potential mechanisms.

From an applied perspective, although subliminal advertisement is illegal in many countries, there is no doubt that many other forms of implicit advertisement are widely used. Subtle or brief product placements can be found in almost all movies, TV sport games, video games, and websites. Our findings suggest that rather than exposing everybody to the same implicit message, tailored messages or product placements that align with people’s personality might be much more effective. The possibility of exposing individuals to implicit advertisements that match their personality is even more relevant given that a lot of personality information is readily available to companies. For example, personality traits can be reliably extrapolated through machine-learning algorithm applied to the configuration of one’s Facebook profile (Back et al., 2010), email box (Back et al., 2008), and even the way one uses his or her phone (de Montjoye et al., 2013).

Our study is among the first to conduct subliminal priming over the Internet (see Weinberger and Westen, 2008; Crump et al., 2013). We readily acknowledge that such method can compromise precision and that it will be important for future research to replicate the present findings in controlled lab settings where a thorough assessment of visibility variables can be conducted. In particular, our open-ended manipulation check cannot completely rule out the possibility that weak conscious processing might have played a role in the present results. Therefore, future research should include a separate objective detection task, not based on subjective judgment but on forced choice to ensure true subliminality (see e.g., Snodgrass et al., 2004; Snodgrass and Shevrin, 2006). Nonetheless, we believe that fast evolution of personal computers over the last decade opens the door to exiting future online research on subliminal priming at a scale never seen before (e.g., individual differences, cultural differences, age differences…). On a less optimistic note, we would also like to highlight that the dangers of mass media subliminal persuasion might be more important than previously thought, as our study demonstrates that non-conscious messages can be successfully processed when broadcasted through the Internet. Thus, it is critical that the ethics of these marketing and persuasion strategies be discussed thoroughly.

Nearly 60 years ago, Vicary’s claim that subliminal injunction to “Drink Coca-Cola” had substantially increased sales of Coca-Cola in a movie theater shocked the public opinion and soon led to legislation prohibiting the use of subliminal communication. Although Vicary’s claims were fabricated, the present research suggests that such legislation may not have been misguided: imagine if Vicary had primed “Drink Red Bull” instead of Coca-Cola, his most impulsive customers might have been tempted to order an energy drink during the intermission...

## Conflict of Interest Statement

The authors declare that the research was conducted in the absence of any commercial or financial relationships that could be construed as a potential conflict of interest.
